# Multiple gating processes associated with the distal end of the S6 segment of domain II in the Nav channels

**DOI:** 10.1016/j.jbc.2024.108060

**Published:** 2024-12-09

**Authors:** Minzhi Chen, Shuijiao Peng, Zhen Xiao, Zhonghua Liu, Xi Zhou

**Affiliations:** 1 The National and Local Joint Engineering Laboratory of Animal Peptide Drug Development, College of Life Sciences, Hunan Normal University, Changsha, China; 2 Peptide and Small Molecule Drug R&D Platform, Furong Laboratory, Hunan Normal University, Changsha, Hunan, China; 3 Institute of Interdisciplinary Studies, Hunan Normal University, Changsha, China; 4 Hunan Provincial Center for Disease Control and Prevention, Hunan Provincial Key Laboratory of Microbial Molecular Biology, Changsha, Hunan, China

**Keywords:** Nav channels, activation gating, inactivation gating, use-dependent slow inactivation, ion selectivity

## Abstract

Voltage-gated sodium (Nav) channels are transmembrane proteins that play crucial roles in the initiation and propagation of action potentials (APs) in excitable tissues such as the heart, muscles, and nerves. The distal ends of the four domain S6 segments of Nav channels contain hydrophobic residues, which form an intracellular gate. This gate allows Nav channels to control ion flux in excitable cells by opening and closing. However, the mechanism of the distal end of Domain II (DII) S6 segment in channel gating remains unclear. In this study, using whole-cell patch clamp recording, we systematically investigated the biophysical characteristics of various mutants L811 site (located at the distal end of the DII S6 segment) of Nav1.9 and the corresponding L796P mutant of Nav1.4. We found that the mutations significantly shifted the activation and inactivation curves, slowed the fast inactivation, accelerated the slow inactivation and use-dependent slow inactivation, and L811P altered the ion selectivity of the channel. Therefore, our findings suggest that the distal end of the DII S6 segment in Nav channels plays a pivotal role in regulating multiple gating processes.

Voltage-gated sodium (Nav) channels are essential for the rapid depolarization phase of action potential (AP) and play a key role in the electrical signaling in most excitable cells (1, 2). Structurally, Nav channels are composed of one pore-forming α-subunit and one or two auxiliary β-subunits (2). The α-subunit is a single polypeptide chain that is composed of four homologous domains (DI to DIV) arranged around a central ion-conducting pore, each domain has a voltage-sensing domain (VSD; S1–S4 segments) and a pore-forming domain (S5–S6 segments) (3, 4). The S4 segments contain repeated motifs of a positively charged amino acid residue (Lys or Arg) followed by two hydrophobic residues, which enable the channel to respond to membrane depolarization and induce S4 outward transfer (3, 5). Eventually, this electromechanical coupling mechanism leads to the iris-like opening of the S6 tetrahelical bundle (5). Therefore, the conformational change of S6 in the four domains controls the opening and closing of the channel pore and plays a vital role in the gating process of the Nav channels.

Accumulating evidence suggests that the S6 segments are the backbone of the ion-conducting pathway, and multiple residues in the S6 segments have been implicated in channel activation and inactivation. Cysteine scanning and accessibility studies suggest that a ring of bulky hydrophobic residues in the lower S6 segments defined the minimal pore gate of Nav channels (6, 7). These residues form an occlusion for ions in the closed state and are splayed open on activation, namely the intercellular gate, which controls channel opening and closing. Many disease-related Nav channel mutations located on the S6 segments impair channel activation and induce certain diseases (7, 8, 9, 10, 11, 12). For example, Nav1.9 mutant L811P, located at the distal end of the S6 segment of DII, causes congenital insensitivity to pain and elicits itch (11, 13). Normally, the Nav channel inactivation gate will occlude the pore after channel opening. Nav channel inactivation is a complex process that includes fast inactivation and slow inactivation (14). Fast inactivation is a hallmark of mammalian Nav channel kinetics and is critical for the repetitive firing of APs. Decades of characterization have established that the short intracellular linker between DIII and DIV (DIII-DIV linker) is a key element for fast inactivation. The key hydrophobic motif Ile-Phe-Met-Thr (the IFMT motif), located in the N-terminal of this linker, is defined as the inactivation particle (15). It is generally believed that the IFMT motif acts as an inactivation particle that enters the intracellular mouth of the pore and blocks the pore when Nav channels are activated (16, 17). However, recent studies in the cryo-EM structures of the Electric Eel or mammalian Nav channels have revealed strikingly similar features, showing that the IFMT motif targets the receptor site formed by amino acid residues in the S4-S5 linker and S6 segments of DIII and DIV, and its repositioning leads to intracellular gate closing through an allosteric effect instead of direct blocking (5, 18, 19). However, the mechanism of DII S6 segment involvement in fast inactivation gating remains unclear.

Alternatively, during prolonged depolarizing proceedings, Nav channels undergo slow inactivation, thereby reducing the number of channels available to provide inward current (20, 21, 22). Therefore, slow inactivation of Nav channels is distinct from fast inactivation and also plays a key physiological role, which has been linked to regulating the excitability of neurons or other excitatory cells (21, 22, 23). Some evidence suggests that it involves conformational rearrangement of the pore domain (24, 25), but the specific mechanism remains unclear.

In this study, we systematically investigated the biophysical characteristics of the clinical mutant L811P of Nav1.9 (this mutant, located at the distal end of the DII S6 segment of Nav1.9, causes pain disorder and itch) (11, 13) and the corresponding L796P mutant of Nav1.4. Our data demonstrate that the L811 mutant significantly alters the activation gating, fast inactivation, slow inactivation gating, and ion selectivity of the channel. Consequently, our results reveal that conformational changes involving the distal end of the DII S6 segment of Nav channels are indispensable for multiple gating processes, including activation, fast inactivation, and slow inactivation gating, and also impact the conformational changes of the selectivity filter.

## Results

### The distal end of the DII S6 segment influences the activation of Nav channels

**Figure 1:**
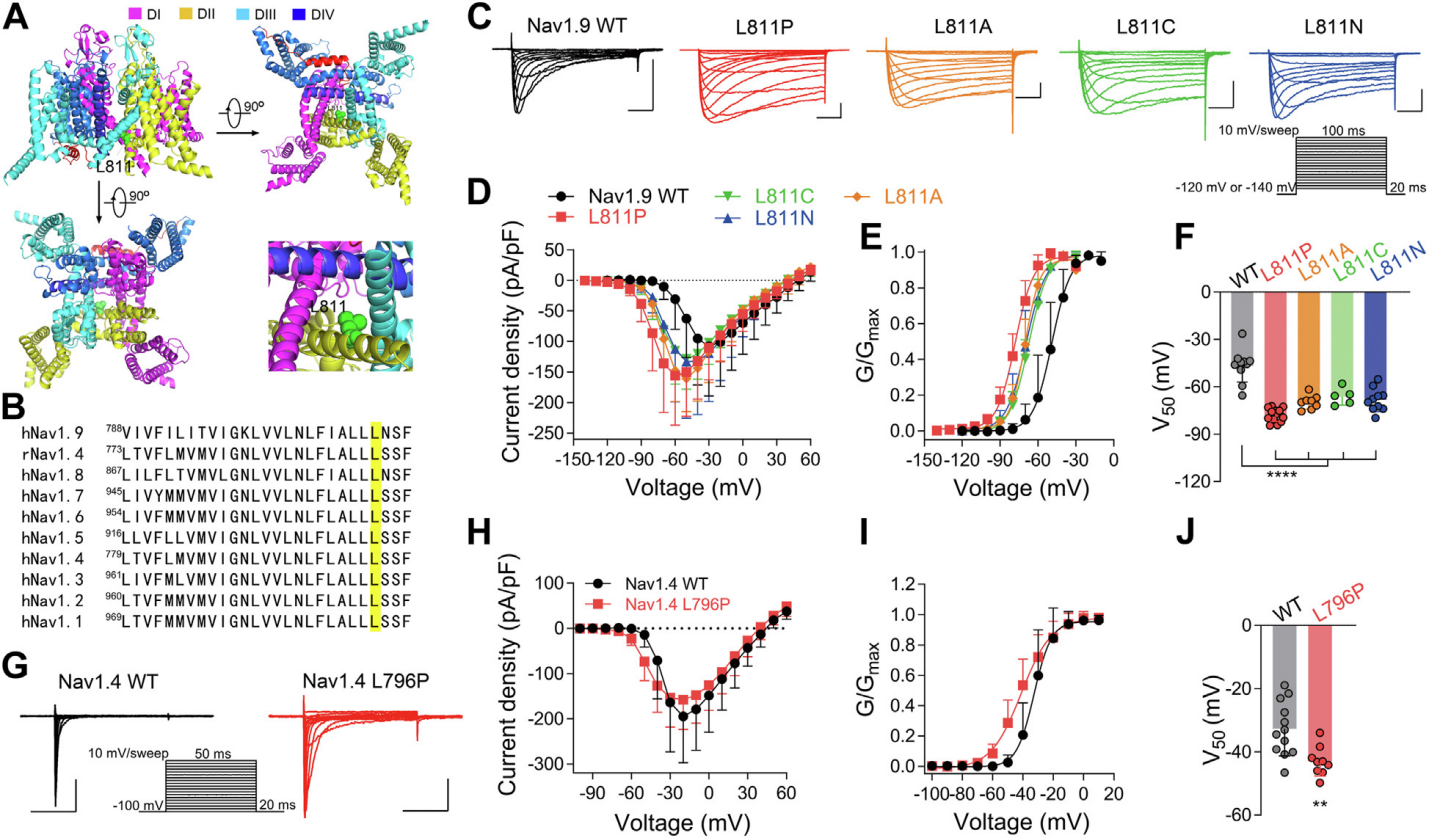
**The Leu site mutation in the distal end of the DII S6 segment hyperpolarized the activation of Nav channel.***A*, the human Nav1.9 structure was predicted by the AlphaFold2 (39) and the structure is domain colored. Three perpendicular views are shown in cartoon, side view (*upper left*), bottom view (*upper right*) and up view (*lower left*). Inset: Intracellular gate (*lower right*). The L811 site is colored green. *B*, sequences alignments corresponding to the DII S6 segment of human Nav channels and rat Nav1.4, the L811 site located the end of DII S6 segment of Nav1.9, it is highly conserved in Nav channels. *C*, representative current traces in ND7/23 cells transfected with Nav1.9 WT (n = 10), L811P (n = 14), L811A (n = 8), L811C (n = 5), and L811N (n = 10), respectively. Scale bar 1 nA, 20 ms. The inset shows the current elicited protocol. *D*, the current density-voltage relationships of Nav1.9 WT and L811 mutations for data in (*C*). *E*, steady-state activation curves fit with a Boltzmann function (Equation 3) (n = 5–14). *F*, scatter dot plot showing the midpoint of activation potential for WT and L811 mutations (one-way ANOVA with *post hoc* analysis using Dunnett's multiple comparisons test, n = 5–14). *G*, representative current traces in HEK293T cells transfected with Nav1.4 WT (*left*, n = 12) and L796P (*right*, n = 9). The inset shows the current elicited protocol. *H*, the current density-voltage relationships of Nav1.4 WT and L796 for data in (*G*). *I*, steady-state activation curves fit with a Boltzmann function (Equation 3) (n = 12 for Nav1.4 WT, n = 9 for L796P). *J*, scatter dot plot showing the midpoint of activation potential for Nav1.4 WT (n = 12) and L796P (n = 9) (two-tailed *t* test). Data are presented as mean ± SD. ∗∗*p* < 0.01, ∗∗∗∗*p* < 0.0001.

In clinical, many disease-related mutations of Nav channels occur in the internal pore region, which significantly affects the gating characteristics of the channels, leading to abnormal channel function and diseases (10, 12). This provides important clues for the study of channel gating dynamics and the relationship between structure and function. Nav1.9 L811P is a previously reported specific *de novo* missense mutation found in individuals with a congenital inability to experience pain (11). The mutation site is located at the distal end of the S6 segment in DII and is conserved in all mammalian Nav channel subtypes (Fig. 1, *A* and *B*), which is close to the intracellular region. The L811P mutant significantly shifts the activation and deactivation kinetics to the left and also slows the fast inactivation (12). These results suggested that L811 in the DII S6 segment is involved in both activation and inactivation gating in the Nav1.9 channel. Therefore, we suggest that this site may be a good sample to investigate the function of the distal end of the S6 segment in DII. In this study, we constructed four mutations including L811P, L811A, L811C, and L811N (Fig. 1*C*), in order to determine whether the L811 site disrupts the multiple gating processes of the channel. The biophysical properties of these four mutants were examined and compared with those of the wild-type (WT) channel. As shown in Figure 1*D*, the mutations evidently increased the current amplitude and negatively shifted both the initial and maximum activation voltages. In addition, the current densities of mutant channels were largely enhanced compared to the WT channel. Compared with the WT, these four mutation channels exhibited a strongly left-shifted voltage dependence of channel activation (Fig. 1*E*). The midpoint of activation for L811P, L811A, L811C, and L811N was significantly shifted by −31.1 mV, −23.0 mV, −19.5 mV, and −21.7 mV (WT = −46.7 ± 10.4 mV; L811P = −77.8 ± 4.1 mV, vs WT *p* < 0.0001; L811A = −69.7 ± 4.4 mV, vs WT *p* < 0.0001; L811C = −66.2 ± 5.5 mV, vs WT *p* < 0.0001; L811N = −68.4 ± 7.3 mV, vs WT *p* < 0.0001), respectively (Fig. 1*F*). However, the slope factors were not significantly different between the mutant and the WT channels (WT = 6.1 ± 1.4 mV, L811P = 6.8 ± 0.9 mV, L811A = 5.8 ± 1.0 mV, L811C = 7.7 ± 2.2 mV, L811N = 6.9 ± 1.2 mV). These results suggest that these mutants favor the open state of the channel, lowering the activation energetic barrier.

To test whether the L811 site has similar functions in different Nav channel subtypes, we mutated the corresponding site, L796, on Nav1.4 to Pro (Fig. 1*B*). It was found that, compared with the WT channel, Nav1.4 L796P also yielded a strongly left-shifted voltage dependence of channel activation, with the activation curve shifting in the hyperpolarizing direction by −10.3 mV (WT = −32.7 ± 8.6 mV, L796P = −43.0 ± 4.7 mV, *p* = 0.004), and significantly altering the slope factor of the steady-state activation curve (WT = 4.6 ± 1.7 mV, L796P = 8.8 ± 0.6 mV, *p* < 0.0001) (Fig. 1, *H*–*J*). Taken together, these results suggest that the distal end of DII S6 segment of the Nav channel affects the voltage dependence of the activation process.

### The distal end of the DII S6 segment influences the fast inactivation of Nav channels

**Figure 2:**
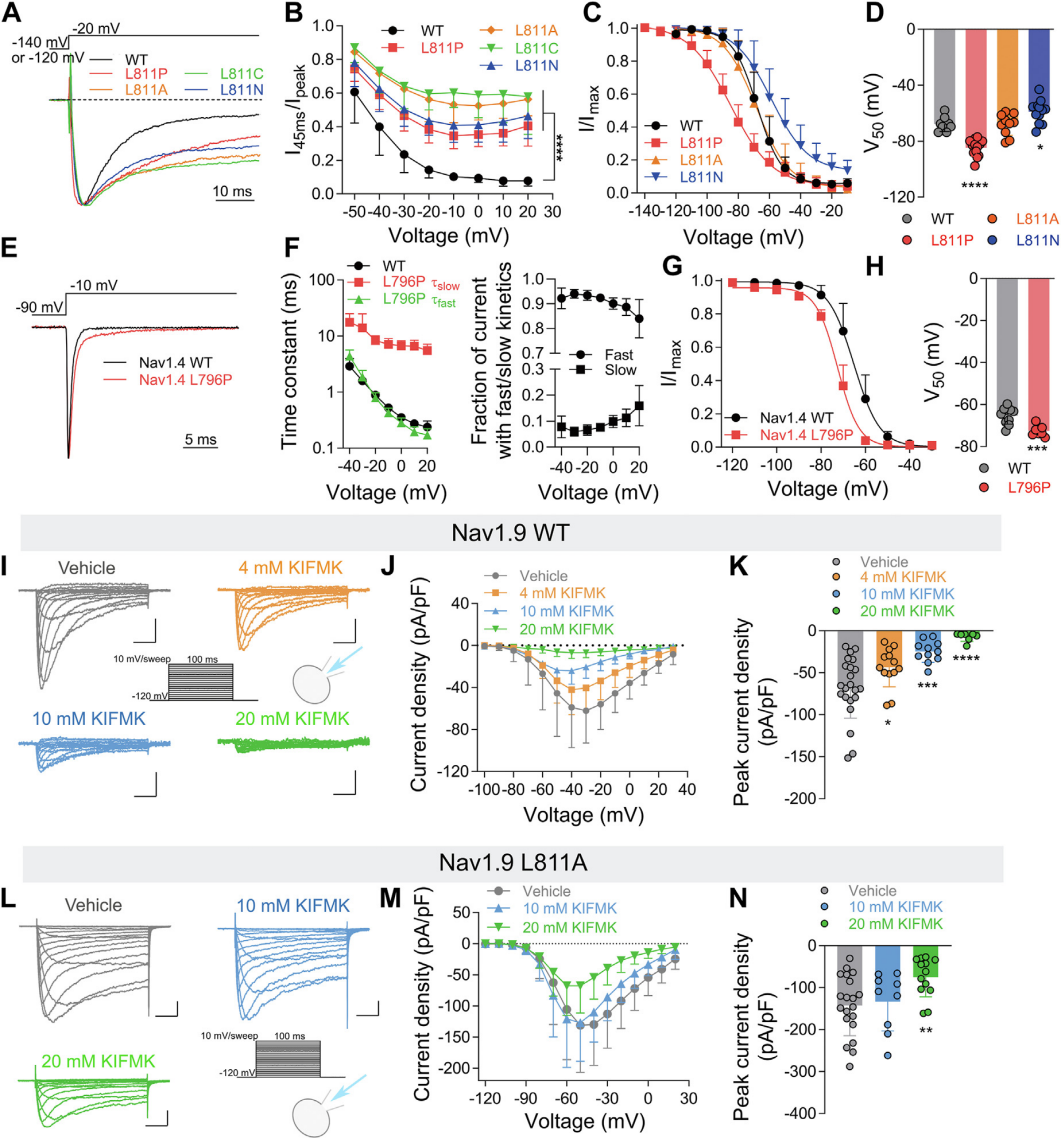
**The L811 mutations make the docking of the IFMT motif with the binding site unstable**. *A*, representative normalized current traces showing fast inactivation kinetics of Nav1.9 L811 mutants compared with Nav1.9 WT (n = 5–14). *B*, L811 mutants significantly enhance the persistent current of the channel compared with Nav1.9 WT (n = 10 for WT, n = 14 for L811P, n = 8 for L811A, n = 5 for L811C, and n = 10 for L811N) (two-way ANOVA followed by Tukey’s multiple comparisons test). *C*, steady-state inactivation of Nav1.9 WT and L811 mutants in ND7/23 cells were measured with a series of 500-ms prepulse (−140 mV or −120 mV to −10 mV in 10-mV increments), followed by a 50-ms depolarization to test potential (−40 mV for WT, L811A and L811N, −60 mV for L811P) to assess the available non-inactivated channels. The ND7/23 cells held at −120 mV (WT, L811A L811C and L811N) or −140 mV (L811P). Data points were well fit with a Boltzmann function (Equation 3) (n = 8 for WT, n = 13 for L811P, n = 10 for L811A, and n = 12 for L811N). *D*, scatter dot plot showing the midpoint of inactivation potential for WT and L811 mutations (one-way ANOVA with *post hoc* analysis using the Dunnett's multiple comparisons test, n = 8–13). *E*, representative normalized current traces during a depolarizing pulse from a holding potential of −90 mV to −10 mV, exhibit significant differences in the fast inactivation kinetics between the Nav1.4 L796P and the Nav1.4 WT. *F*, *Left panel*, time constant of fast inactivation for Nav1.4 WT and L796P. Single-exponential decay current time constants as a function of voltage for Nav1.4 WT (n = 8), double-exponential decay current time constants as a function of voltage for Nav1.4 L796P (n = 7). *Right panel*, the fractions of fast- and slow-inactivating components of Nav1.4 L796P currents. *G*, Steady-state inactivation of Nav1.4 WT and L796P in HEK293T cells were measured with a series of 500-ms prepulse (−120 mV to −30 mV in 10-mV increments), followed by a 50-ms depolarization to 0 mV to assess the available non-inactivated channels. The HEK293T cells held at −120 mV. Data points were well fit with a Boltzmann function (Equation 3) (n = 10 for WT and n = 8 for L796P). *H*, Scatter dot plot showing the midpoint of inactivation potential for Nav1.4 WT (n = 10) and L796P (n = 8) (two-tailed *t* test). *I–N*, the effect of intracellularly applied KIFMK peptide on the Nav1.9 WT (*I–K*) and L811A (*J–L*) channels in ND7/23 cells. Representative current traces in the absence or presence of KIFMK peptide at different concentrations are shown for the Nav1.9 WT (*I*) and L811A (*L*) channels. Scale bar 1 nA, 20 ms. The inset shows the current elicited protocol. Applying the KIFMK peptide at varying concentrations intracellularly affects the current density of the Nav1.9 WT (*J*, n = 7–23 for per group) and L811A (*M*, n = 9–20 for per group). Scatter dot plot showing the effect of KIFMK peptide on the peak current density of Nav1.9 WT (*K*, one-way ANOVA with *post hoc* analysis using the Dunnett's multiple comparisons test, n = 7–23 for per group) and L811A (*N*, one-way ANOVA with *post hoc* analysis using the Dunnett's multiple comparisons test, n = 9–20 for per group). Data are presented as mean ± SD. ∗*p* < 0.05, ∗∗*p* < 0.01, ∗∗∗*p* < 0.001, ∗∗∗∗*p* < 0.0001.

Fast inactivation is an important gating characteristic of Nav channels, we next determined whether the distal end of the DII S6 segment is also involved in the fast inactivation process. Compared to the WT channel, L811 mutants significantly slowed the fast inactivation of channels. We found that all four L811 mutants produced a very large persistent current (Fig. 2*A*). Within the stimulation pulse ranging from −10 mV to 20 mV, 40%-60% of the current remained in L811 mutants relative to the peak current at the end of 45 ms depolarization, while the WT channel was almost completely inactivated (Fig. 2*B*). Furthermore, we tested whether the mutants affected the voltage dependence of inactivation. We categorized the effects of mutations on the voltage dependence of inactivation as left-shift, right-shift, and no-effect mutants. Figure 2, *C* and *D* show the mutant L811P yielded a strongly left-shifted voltage dependence of channel inactivation (WT = −67.8 ± 5.3 mV, L811P = −84.8 ± 6.0 mV, vs WT *p* < 0.0001). The L811N mutant showed a right shift by 8.9 mV (L811N = −58.9 ± 8.2 mV, vs WT *p* = 0.014), while L811A (L811A = −68.2 ± 7.7 mV, vs WT *p* = 0.92) displayed no change in the voltage dependence of the inactivation. However, the slope factors were significantly different between the mutation and WT channels (WT = 7.3 ± 1.0 mV; L811P = 13.3 ± 1.7 mV, vs WT *p* < 0.0001; L811A = 9.7 ± 1.5 mV, vs WT *p* = 0.017; L811N = 10.6 ± 2.2 mV, vs WT *p* = 0.0004). These results indicate that the L811 site of Nav1.9 is involved in the fast inactivation process of the channel. The same results were also observed in the Nav1.4 L796P channel, as shown in Figure 2*E*. The Nav1.4 L796P mutant significantly delayed the fast inactivation of the channel and altered the kinetics of fast inactivation. The fast inactivation of the WT channel fits a single exponential equation, whereas the fast inactivation of Nav1.4 L796P fits a double exponential equation. Compared with the WT, L796P showed a similar τ_fast_ but exhibited a large τ_slow_ at −40 to +20 mV (Fig. 2*F* *left panel*). Moreover, the fraction of the slow-inactivating component progressively increases from −30 to +20 mV (Fig. 2*F* *right panel*). Therefore, the slower τ_slow_ of L796P may be the reason for the slower fast inactivation of the channel. Next, we measured the effect of L796P on voltage-dependent inactivation and found that, similar to Nav1.9 L811P, the Nav1.4 L796P mutation also shifted the voltage-dependent inactivation towards the hyperpolarizing direction, with the midpoint of inactivation for L796P significantly shifted by −7.3 mV (WT = −65.2 ± 4.1 mV, L796P = −72.5 ± 2.5 mV, *p* = 0.0004), and the slope factor was changed slightly (WT = 5.2 ± 0.5 mV, L796P = 4.5 ± 0.5 mV, *p* = 0.008) (Fig. 2, *G* and *H*).

The fast inactivation of Nav channels occurs due to the binding of the “inactivation ball”, the IFMT motif, located in the intracellular linker domains of DIII and DIV, to its receptor site, leading to pore closure (3, 15). We aimed to investigate whether the L811 mutation affects the binding of the IFMT motif to its receptor site. We synthesized the KIFMK peptide (a free peptide that mimics the binding of the IFMT motif to the receptor site) and determined its inhibitory effect on Nav currents (26, 27, 28). When added to the intracellular solution, the KIFMK peptide inhibited Nav1.9 WT currents in a concentration-dependent manner, with 10 mM KIFMK peptide inhibiting approximately 63.4% of Nav1.9 WT currents and 20 mM KIFMK peptide almost completely inhibiting them (Fig. 2, *I*–*K*). However, the KIFMK peptide had a reduced effect on Nav1.9 L811A mutant currents; 10 mM KIFMK peptide showed no significant inhibition of Nav1.9 L811A mutant currents, and increasing the KIFMK peptide concentration to 20 mM only inhibited approximately 47.0% of the currents (Fig. 2, *L*–*N*). These results indicate that the L811A mutation may decrease the binding of the IFMT motif to its receptor site, suggesting that the mutation may affect the conformation of the IFMT motif binding site, making the docking of IFMT motif with the binding site unstable and reducing their affinity.

### L811P promotes the slow inactivation

**Figure 3:**
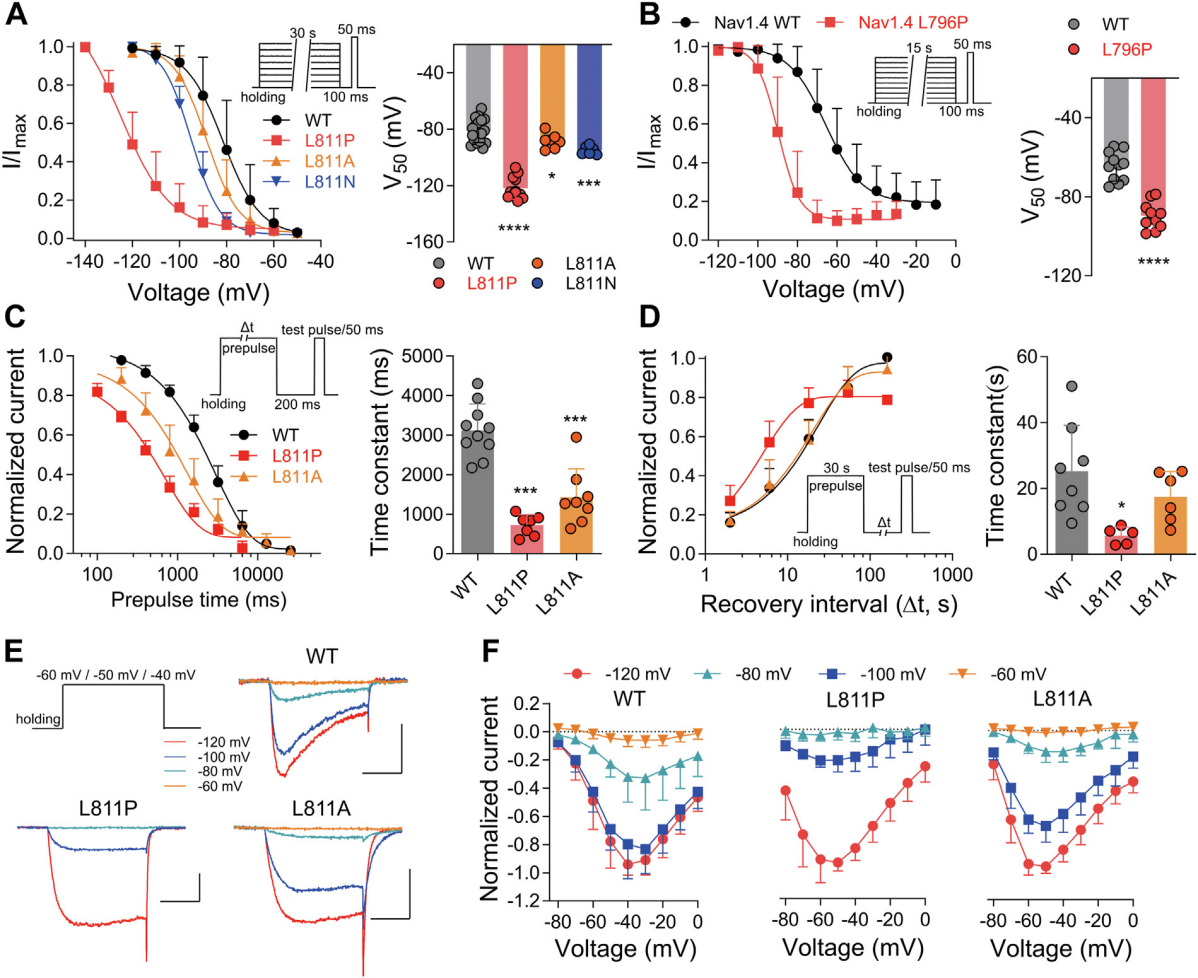
**L811P accelerates the slow inactivation.***A*, the steady-state slow inactivation curves of Nav1.9 WT and L811 mutations, data points for slow inactivation kinetics were well fitted with the Boltzmann equation (Equation 3, *left*). Scatter dot plot showing the midpoint of slow inactivation potential for WT and L811 mutations (*right*) (n = 19 for WT, n = 12 for L811P, n = 7 for L811A, and n = 6 for L811N) (one-way ANOVA with *post hoc* analysis using the Dunnett's multiple comparisons test). The slow inactivation was measured with a series of 30-s prepulses (−140 or −120 mV to −50 mV in 10-mV increments), followed by a 100-ms pulse to the holding potential to remove fast-inactivation, and then depolarization to −60 mV (L811P) or −40 mV for a duration of 50-ms (WT, L811A and L811N) to assess the available non-inactivated channels. The ND7/23 cells held at −120 mV (WT, L811A and L811N) or −140 mV (L811P). *B*, the steady-state slow inactivation curves are shifted −25 mV by Nav1.4 L796P (*left*). Scatter dot plot showing the midpoint of slow inactivation potential for WT and L796P (*right*) (n = 11 for WT and n = 10 for L796P) (two-tailed *t* test). The inset shows the current elicited protocol, and the HEK293T cells held at −120 mV. *C*, kinetics of onset of slow inactivation show the L811P and L811A mutants exhibited accelerated slow inactivation (*left*). The inset shows the current elicited protocol, and the ND7/23 cells held at −120 mV (WT and L811A) or −140 mV (L811P). Comparison of the onset time constants of Nav1.9 WT (n = 10), L811P (n = 7) and L811A (n = 8) (*right*) (one-way ANOVA with *post hoc* analysis using the Dunnett's multiple comparisons test). *D*, kinetics of recovery from slow inactivation show accelerated recovery for the L811P (*left*). The inset shows the current elicited protocol, and the ND7/23 cells held at −120 mV (WT and L811A) or −140 mV (L811P). Comparison of the recovery time constants of Nav1.9 WT (n = 8), L811P (n = 5) and L811A (n = 6) (*right*) (one-way ANOVA with *post hoc* analysis using the Dunnett's multiple comparisons test). *E* and *F*, representative current traces were activated by a 50-ms depolarization to −40 mV (WT), −50 mV (L811A) or −60 mV (L811P) from a holding potential of −120 mV (*red*), −100 mV (*blue*), −80 mV (*cyan*) or −60 mV (*orange*), respectively (*E*). Scale bar 1 nA, 20 ms. *F*, current-voltage relationships measured for the different holding potentials. Current amplitudes were normalized to the maximum peak current measured at the holding potential of −120 mV for each channel. Data are presented as mean ± SD. ∗*p* < 0.05, ∗∗∗*p* < 0.001, ∗∗∗∗*p* < 0.0001.

Slow inactivation in Nav channels is an important biophysical process that governs their availability over extended periods of time and can function as a brake during periods of neuronal hyperexcitability (22, 23). The slow inactivation of Nav channels may involve the rearrangement of the pore domain (25, 29). Next, we measured the steady-state slow inactivation of the L811 mutants. Compared with the WT channel, they exhibited a significant negative shift in slow inactivation (WT = −81.3 ± 8.1 mV; L811P = −121.9 ± 7.9 mV, vs WT *p < 0.0001*; L811A = −88.3 ± 5.3 mV, vs WT *p = 0.046;* L811N = −95.0 ± 3.0 mV, vs WT *p = 0.0006*) (Fig. 3*A*). Similarly, as shown in Figure 3*B*, Nav1.4 L796P also shifts the steady-state slow inactivation curve towards the hyperpolarized direction (WT = −64.7 ± 7.4 mV; L796P = −89.7 ± 7.0 mV, *p < 0.0001*). Furthermore, the onset of and recovery from slow inactivation were also examined in Nav1.9 WT, L811P, and L811A, revealing that both processes were accelerated in the L811P mutant compared to the WT channel (*onset*, WT: τ_onset_ = 3.12 ± 0.67 s; L811P: τ_onset_ = 0.72 ± 0.26 s, *p < 0.0001*; *recovery*, WT: τ_recovery_ = 25.3 ± 13.9 s; L811P: τ_recovery_ = 5.7 ± 2.6 s, *p = 0.01*), whereas the L811A mutation accelerated the onset of slow inactivation (L811A: τ_onset_ = 1.43 ± 0.72 s, *p* < 0.0001) but had no effect on its recovery (L811A: τ_recovery_ = 17.4 ± 7.7 s, *p = 0.24*) (Fig. 3, *C* and *D*). Note that only 80% of the current was recovered for the L811P mutant upon prolonged recovery duration, whereas complete recovery could be achieved for the WT channel and L811A. We propose that the accelerated slow inactivation caused by the L811P and L811A mutations would reduce channel availability. As shown in Figure 3, *E* and *F*, with increasing holding potential, the current amplitude of the L811P and L811A mutations decreased sharply, with only 3.9 ± 4.4% and 15.6 ± 7.6% current remaining at −80 mV, respectively. Correspondingly, 36.8 ± 21.9% of the current was retained for the WT channel. Taken together, these results suggest that the distal end of DII S6 segment L site may play a key role in the slow inactivation of Nav channels.

### The distal end of the DII S6 segment affects the use-dependent inactivation of Nav channels

**Figure 4:**
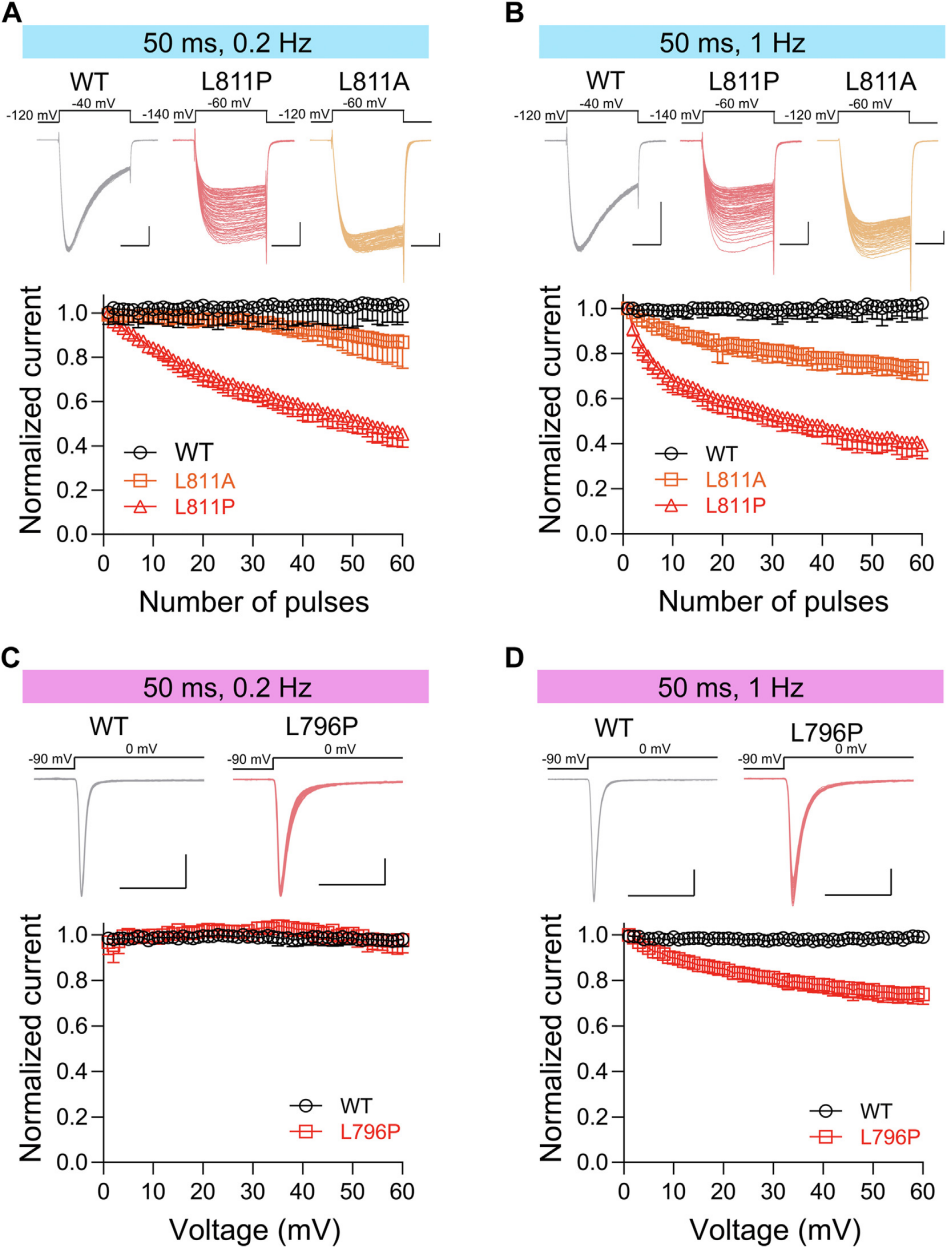
**The Leu site mutation in the distal end of the DII S6 segment accelerates the use-dependent inactivation of Nav channels.***A* and *B*, *Upper*, representative Na^+^ currents elicited in ND7/23 cells expressing the WT (*black*), L811P (*red*) or L811A (*orange*) hNav1.9 channel by a 0.2 Hz (*A*, n = 4 for WT, n = 4 for L811P and n = 5 for L811A) and 1 Hz (*B*, n = 4 for WT, n = 4 for L811P and n = 5 for L811A) pulse train. The cells, held at −120 mV (WT and L811A) or −140 mV (L811P), were depolarized to −40 mV (WT) or −60 mV (L811P and L811A) for 50-ms repetitive pulses applied at 0.2 Hz (*A*) or 1 Hz (*B*). Scale bar 1 nA, 20 ms. *Lower*, the current amplitudes are normalized to those elicited by the first pulse. *C* and *D*, *Upper*, representative Na^+^ currents elicited in HEK293T cells expressing the WT (*black*) or L796P (*red*) Nav1.4 channel by a 0.2 Hz (*C*, n = 7 for WT, n = 3 for L796P) and 1 Hz (*D*, n = 7 for WT, n = 3 for L796P) pulse train. The cells, held at −90 mV were depolarized to 0 mV) for 50-ms repetitive pulses applied at 0.2 Hz (*C*) or 1 Hz (*D*). Scale bar 1 nA, 20 ms. *Lower*, the current amplitudes are normalized to those elicited by the first pulse. Data are presented as mean ± SD.

Here, under different frequency repetitive stimulations, we revealed that, in contrast to the WT channel, the L811P and L811A mutants displayed gradual decay of currents evoked at 0.2 Hz (Fig. 4*A*) and 1 Hz (Fig. 4*B*). Repetitive depolarizations of Nav1.9 WT to −30 mV for 50 ms at either 0.2 Hz or 1 Hz showed no use-dependent inactivation. However, after 60 pulses at 0.2 Hz and 1 Hz, respectively, L811P resulted in a reduction of current amplitude to 55 ± 5.9% and 60.7 ± 5.9% of its initial value. Furthermore, under the high-frequency stimulation of 1 Hz, the decay speed of the L811P current is faster than under low-frequency stimulation of 0.2 Hz (Fig. 4, *A* and *B*). This phenomenon is also found in L811A mutant, but compared with L811P, the current decay of L811A is lower under both 0.2 Hz and 1 Hz. By the end of 60 pulses, L811A reduced its current amplitude to only 73.4 ± 5.3% of its initial value at 1 Hz compared with a 39.3 ± 5.9% reduction in the case of L811P (Fig. 4, *A* and *B*). These results are consistent with the effects of mutants on slow inactivation, which leads to a greater hyperpolarization shift of the slow inactivation curve and accelerates the entry into slow inactivation of L811P, resulting in greater use-dependent inactivation. Furthermore, in the Nav1.4 channel, we observed that under 1 Hz high-frequency repetitive stimulation, L796P reduced the current amplitude to roughly 26.1 ± 4.3% of its initial value after 60 pulses, whereas the WT channel did not exhibit use-dependent inactivation (Fig. 4, *C* and *D*). These findings highlight the impact of the distal end of the DII S6 segment on the use-dependent inactivation of Nav channels.

### L811P mutant alters the ion selectivity of the channel

**Figure 5:**
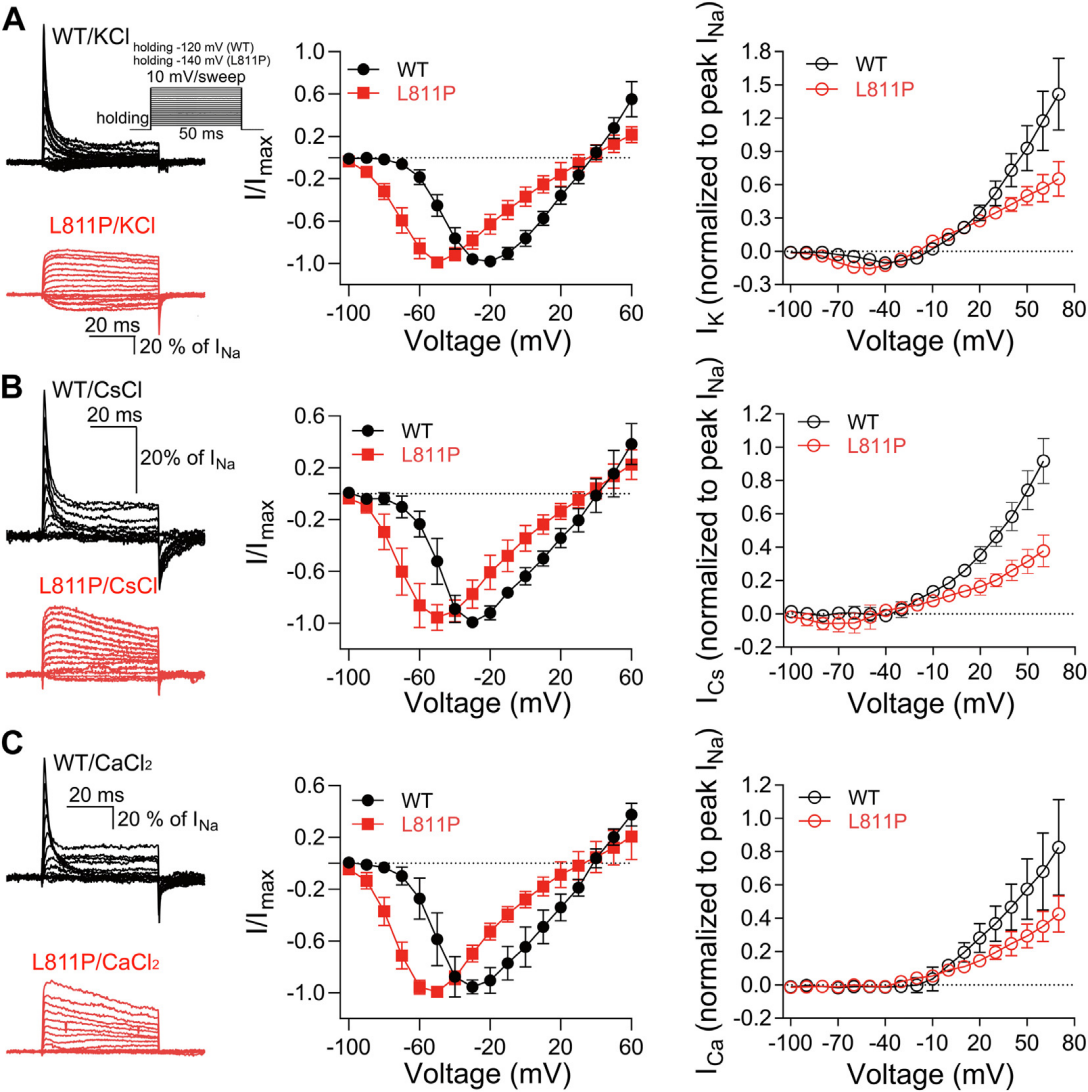
**L811P alters the permeabilities of channels to K**^**+**^**, Cs**^**+**^**, and Ca**^**2+**^. *A*–*C*, from *top* to *bottom*, the *left* panels show representative current traces from WT and L811P channels in KCl- (*A*), CsCl- (*B*), and CaCl_2_-based (*C*) extracellular solutions in CHO-K1 cells, the inset shows the current evoked protocol. For WT, the holding potential is −120 mV. For L811P, the holding potential is −140 mV. The middle panels show the I-V curves for WT and L811P in the NaCl-based external solution before substituting K^+^, Cs^+^ or Ca^2+^. The *right panels* show I-V relationships for WT and L811P channels in KCl- (*A*, n = 7 for WT, n = 11 for L811P), CsCl- (*B*, n = 7 for WT, n = 7 for L811P), and CaCl_2_-based (*C*, n = 9 for WT, n = 8 for L811P) solutions. Data are presented as mean ± SD.

As shown in Figure 1*D*, the L811 mutations caused a notable alteration, specifically an approximately −5 mV shift in the reversal potential (from 50 mV for WT to 45 mV for L811P) which was probably caused by ion selectivity change. Cheng *et al.* reported that a deletion mutation of Nav1.7 (Del-L955, corresponding to site L809 in Nav1.9) shifted reversal potential by approximately −7 mV, and they further revealed that the Del-L955 mutation increased permeabilities to Cs^+^ and Ca^2+^ (30). Therefore, we further investigated whether the L811P mutant also alters the ion selectivity. Figure 5*A* shows the permeability of WT and L811P to K^+^. On the left are the raw traces from cells expressing WT and L811P in K^+^-based extracellular solution. Both WT and mutant channels allow K^+^ to pass through the channel pore. We subsequently compared the permeabilities to K^+^ and Na^+^ by conducting measurements in NaCl-based and KCl-based solutions, respectively. The right panel displays the current-voltage (I-V) curves, normalized to the maximal inward transient peak currents in NaCl-based solution, for KCl-based solution. Consistent with our results, L811P hyperpolarized shifts in activation (WT = −47.3 ± 4.3 mV, L811P = −64.2 ± 6.7 mV, two tail *t* test, *p* = 0.0002) and reversal potential. The maximal I_K_/I_Na_ ratios of WT and L811P channels are 10.6 ± 0.9% (percentage of the maximal inward transient peak current in NaCl-based solution) and 16.2 ± 0.7% (two-tailed *t* test, *p* = 0.0001), respectively. The reversal potential (corrected by liquid junction potential) is −9.7 ± 4.9 mV for WT and −22.4 ± 6.9 mV for L811P channels (two-tailed *t* test, L811P vs WT, *p* = 0.0007). The P_K_/P_Na_ ratio of L811P channels was not significantly different from that of WT (L811P = 0.17 ± 0.03, WT = 0.15 ± 0.04, two-tailed *t* test, *p* = 0.25). However, as shown in Figure 5*B*, when cells were replaced by CsCl-based extracellular solutions, micro inward currents were observed from L811P channels (The maximal I_Cs_/I_Na_ is 0.06 ± 0.04), whereas no inward currents were detected from WT channels. The P_Cs_/P_Na_ ratio of L811P channels is 0.03 ± 0.01. As shown in Figure 5*C*, when cells were replaced by CaCl-based extracellular solutions no inward currents were detected from WT and L811P channels. Taken together, the L811P mutation alters the ion selectivity by enhancing the permeability of the channels to Cs^+^.

## Discussion

The pore-forming α-subunit of the Nav channels comprises four homologous domains (DI–IV). The S5 and S6 helices of each domain, along with a membrane-reentrant pore loop (P-loop) between S5 and S6 form the ion-conducting pore. The S6 helices constitute the backbone of the ion-conducting pathway and are important for channel activation and inactivation gating. Here, we revealed that the distal end of the DII S6 segment of the Nav channel is coupled with multiple gating processes, including activation, fast inactivation, and slow inactivation gating, and also affects the conformational changes of the selectivity filter.

The distal ends of the four domain S6 helices contain hydrophobic residues that form an intracellular activation gate, regulating Na^+^ passage upon membrane potential depolarization. Accessibility analyses have identified four hydrophobic residues at homologous positions in each domain of Nav1.4, which form an occlusion for ions in the closed state and are splayed open on activation (6). According to the sequence alignment between Nav1.4 and Nav1.9 (Fig. 1*B*), for Nav1.9, L810 of DII and the paralogous positions in the other three domains shape the activation gate. Additionally, these residues form the docking site for the fast inactivation ball after channel activation, which is critical for fast inactivation (31). Slow inactivation is the process associated with pore structure arrangement, requiring conformational changes that occur both at the P-loops and S6 distal end. Therefore, it is not surprising that amino acid substitutions at the S6 distal end cause evident changes in channel gating. Actually, alanine substitutions at the distal ends of DI and DII S6 segments cause significant changes in Nav1.2 channel activation (32), and several mutations occurring at the distal end of Nav1.4 S6 segment impair its slow inactivation (31, 33, 34). In particular, the L811P mutation modifies Nav1.9 activation, fast activation, slow inactivation, and ion selectivity, as revealed in this work. It may not only affect the distal end of the DII S6 segment but also influence allosterically the structures of neighboring helices, which likely impairs the integrity of the activation gate and the docking site for fast inactivation, as well as dynamic conformational change during channel gating. Furthermore, this mutation appears to affect the conformational change of the ion selectivity filter during channel gating, since it evidently alters the ion selectivity of Nav1.9. On the other hand, our study indicated that the L811A, L811C, and L811N substitutions actually lead to some different effects on Nav1.9 channel gating, such as for voltage-dependent fast inactivation (Fig. 2, *C* and *D*). Additionally, we found that the L810P and L809P mutations severely compromise functional channel expression in ND7/23 cells, which suggests that this region might be crucial to the structure and function of Nav1.9. These data suggest that the structural integrity of DII S6 segment, rather than a single amino acid change in this region, is responsible for the robust change of channel gating.

The L811P mutant channel has been studied intensively, but the basis of the fundamental difference between functional data and the clinical outcome remains elusive (8, 11). The L811P mutant channel activates at a more hyperpolarized potential and shows a larger current density than Nav1.9 WT. In this regard, it is reasonable to consider this mutant to be gain-of-function. However, according to our data, this mutant demonstrates some distinct biophysical properties from other gain-of-function mutants causing increased pain: (i) it shifts Nav1.9 fast and slow inactivation by −17 mV and −40.6 mV, respectively, as well as results in a significant more rapid conversion to the slow inactivation state, which drives Nav1.9 channels in the cell surface to be more likely to undergo the inactivated state and consequently reduces their availability even at resting membrane potential; (ii) it alters the ion selectivity through the pore and the reversal potential, and consequently mediates large and sustained outward currents, causing neurons to repolarize rather than depolarize at more depolarized potentials and therefore decreasing neuron excitability; (iii) it causes Nav1.9 current decay sharply at sustained repetitive firing, which might be interpreted as loss of channel availability of Nav1.9 in this condition (8). Nav1.9 is a key contributor to the initiation and frequency of AP during repetitive firing (35, 36). The rapid decay of the L811P mutant currents might provide an explanation that the L811P mutant associates with significantly reduced frequency and gradual loss of APs during extended firing periods and then dampens neuron excitability (8). In general, these distinct attributes of the L811P mutant might prevail over the effect of enhanced activation at pathological pain conditions and consequently result in a painless phenotype of clinical outcome.

## Experimental procedures

### Plasmid constructs and mutagenesis

The deleted stop codon cDNA of human Nav1.9 (hNav1.9), using KpnI and SmaI enzyme site, was subcloned into the pEGFP-N1 vector (Clontech), which was as described in our previous studies (37). Rat Nav1.4 cDNA clones were subcloned into the pCDNA3.1-blank vectors. Mutations were constructed by using the Quick-change II XL Site-directed Mutagenesis kit (Agilent Technologies) according to the manufacturer’s instruction. All mutations were verified by DNA sequencing.

### KIFMK peptide synthesis and purification

KIFMK peptide synthesized manually using a Fmoc [N-(9-fluorenyl) methoxycarbonyl]/tert-butyl strategy and HOBt/TBTU/NMM coupling method. Synthetic crude products were purified by reverse-phase high-performance liquid chromatography (RP-HPLC) using an Ultimate XB-C18 column (300 Å, 10 mm × 250 mm, Welch Materials Inc.) on the Hanbon HPLC system (Hanbon Sci&Tech). A linear gradient of solvent A (0.1% trifluoroacetic acid (TFA) in acetonitrile) in solvent B (0.1% TFA acid in water) was employed at a flow rate of 3 min/ml: starting with 5% A for 5 min, followed by a gradient increase to 35% A over 30 min. Absorbance was monitored at 215 nm. The molecular weights of KIFMK peptide were determined using matrix-assisted laser desorption/ionization-time-of-flight mass spectrometry (MALDI–TOF-TOF MS) (AB SCIEX TOF/TOF 5800 system, Applied Biosystems).

### Cell culture and transfection

ND7/23 and HEK293T cells were maintained at 37 °C in a humidified 5% CO_2_ incubator in Dulbecco’s Modified Eagle’s Medium (DMEM) supplemented with 10% fetal bovine serum (FBS), 100 μg/ml streptomycin, 100 U/ml penicillin, and 2 mM Lglutamine. CHO-K1 cells were maintained in DMEM-F12K mixed medium (1:1) with 10% FBS and cultured under the same conditions. These cell lines were purchased from Stem Cell Bank, Chinese Academy of Sciences. Transient transfection of Nav1.4 or L796P channel plasmids was cotransfected with eGFP into HEK293T cells using Lipofectamine 2000 (Thermo Fisher Scientific) according to the manufacturer’s instructions. The ND7/23 or CHO-K1 cells were transfected with hNav1.9 or L811 mutations using the X-tremeGENE HP DNA Transfection Reagent (Roche, Basel, Switzerland) according to the manufacturer’s instructions. Cells with green fluorescent protein (GFP) were selected for whole-cell patch-clamp analysis at 24 to 36 h post-transfection.

### Electrophysiological

Whole-cell patch-clamp recordings were conducted using an EPC-10 USB patch-clamp amplifier operated by PatchMaster software (HEKA Elektronik, Lambrecht, Germany). Fire-polished electrodes (2.0–2.5 MΩ) were prepared in a PC-10 puller (NARISHIGE, Tokyo, Japan) using a two-step program. Capacity transients were canceled, and series resistance was maintained below 10 MΩ with voltage errors minimized by 80% series resistance compensation in the whole-cell configuration. Recordings were performed at room temperature (25 ± 2 °C). For recording Nav channels, the bath solution contains (in mM): 150 NaCl, 2 KCl, 1.5 CaCl_2_, 1 MgCl_2_, 10 HEPES, and 10 Glucose (pH 7.4 with NaOH), and the pipette solution contains (in mM): 105 CsF, 35 NaCl, 10 EGTA and 10 HEPES (pH 7.4 with CsOH). The osmotic pressure of the intracellular fluids and extracellular fluids is adjusted to 300 to 320 mOsm with sucrose. For electrophysiology experiments, the stock solution of the KIFMK peptide was diluted with fresh pipette solution to a final concentration suitable for intracellular application *via* the pipette. The voltage-dependent currents were acquired at least 5 min after establishing a whole-cell configuration. Additionally, 1 μM tetrodotoxin (TTX) was supplemented in recordings of Nav1.9 and L811 mutation channels to inhibit endogenous TTX-sensitive (TTX-S) Nav channel in ND7/23 cells.

To generate activation curves, cells were held at −120 mV (Nav1.9 W T，L811A, L811C and L811N) or −140 mV (L811P) or −100 mV (Nav1.4 WT and L796P) and stepped to +50 mV in 10-mV increments from the holding potential for 50-ms every 5-s. The G-V curves were obtained by calculating the conductance (*G*) at each voltage (*V*) using the equation:

(1) $$G=\frac{I}{V-V_{rev}}$$

Where, *I* is the peak current, *V* is the corresponding step voltage and *V*_*rev*_ is the reversal potential determined for each cell individually.

G-V curves were fitted using a Boltzmann equation:

(2) $$y=\frac{1}{1+e^{\left(V_{50}-V\right)/\kappa }}$$

Where, *V*_*50*_, *V*, and κ represented the midpoint voltage of kinetics, test potential and slope factor, respectively. Nav1.9 WT and L811 mutants were expressed in ND7/23 cells, and Nav1.4 WT and L796P were expressed in HEK293T cells.

Voltage-dependent steady-state inactivation was measured using a series of 500-ms pre-pulses ranging from −120 mV or −140 mV to −10 mV in 10-mV increments, followed by a 50-ms depolarization to −40 mV (for Nav1.9 WT, L811A and L811N), −60 mV (for Nav1.9 L811P), or −0 mV (for Nav1.4 WT and L796P) to assess the available non-inactivated currents. The repetition interval was 10-s for Nav1.9 WT and mutants, and 5-s for Nav1.4 WT and L796P. Steady-state slow-inactivation was using 30-s pre-pulses (for Nav1.9 WT and mutants) or 15-s pre-pulses (for Nav1.4 WT and L796P) ranging from the holding potential to −50 mV or −10 mV. This was followed by a 100-ms pulse to the holding potential to remove fast inactivation. The remaining available channels were activated by a 50-ms test pulse to −40 mV (for Nav1.9 WT, L811A and L811N), −60 mV (for Nav1.9 L811P), or 0 mV (for Nav1.4 WT and L796P). The repetition interval was 40-s. The holding potential is −140 mV for L811P, −120 mV for Nav1.9 WT, L811A, and L811N, and −120 mV for Nav1.4 WT and L796P. Peak inward currents at the test pulse were normalized to the maximal inward current and fit with a Boltzmann function:

(3) $$I/I_{\max}=A+\frac{1-A}{1+e^{\left(V-V_{50}\right)/\kappa }}$$

Where, *V* represents the inactivating pre-pulse potential, *V*_*50*_ is the midpoint of the steady-state inactivation or slow inactivation, *A* is the minimal channel availability, and *κ* is the slope factor. Nav1.9 WT and L811 mutants were expressed in ND7/23 cells, and Nav1.4 WT and L796P were expressed in HEK293T cells.

To measure the kinetics of onset of slow inactivation, the ND7/23 cells membrane was depolarized to −50 mV (for Nav1.9 L811P) or −40 mV (for Nav1.9 WT and L811A) for durations that varied from 0 to 25.6-s, followed by repolarization to the holding potential for 200-ms and by a 50-ms depolarizing test pulse to −50 mV or −40 mV. The repetition interval was 40-s. To measure the rates of recovery from slow inactivation, the cells membrane was depolarized to −50 mV (for Nav1.9 L811P) or −40 mV (for Nav1.9 WT and L811A) for 30-s followed by a repolarization to the holding potential for 2 to 162-s and by a test pulse to −50 mV or −40 mV for 50-ms. The repetition interval was 40-s. The kinetics of onset of slow inactivation and the rates of recovery of slow inactivation were fitted with a single exponential function:

(4) $$y=y0+a\left(1-e^{-x/\tau }\right)$$

Where, τ represents the time constant.

### Ion selectivity

To investigate the effect of L811P on channel permeability to Cs^+^, K^+^, and Ca^2+^, we referred to the methodology employed by Cheng *et al.* (30). To avoid the influence of endogenous Nav channels, voltage-gated potassium channels, and calcium channels present in ND7/23 cells, CHO-K1 cells were specifically chosen to express Nav1.9 WT and its mutant L811P, solely for the purpose of investigating ion selectivity. Briefly, the pipette solution contained (in mM): 140 CsF, 10 NaCl, 1 EGTA, 10 glucose, and 10 HEPES, pH 7.3 with CsOH. The NaCl-based bath solution contained (in mM): 140 NaCl, 3 KCl, 1 MgCl_2_, 1 CaCl_2_, 10 glucose, 10 HEPES, pH 7.34 with NaOH. For tested cation solutions, the 140 mM NaCl in NaCl-based bath solution was replaced by 140 mM CsCl, or 140 mM KCl (total KCl 143 mM), or 100 mM CaCl_2_ (total CaCl_2_ 101 mM), and pH was adjusted to 7.3 with Tris-base. The osmolarity of all solutions was maintained at 300 to 320 mOsm by sucrose.

Permeabilities of WT and L811P channels to K^+^, Cs^+^, and Ca^2+^ were examined using whole-cell patch-clamp recording. Cells were first incubated in a tested cation-based bath solution and the current-voltage (I-V) curve was recorded. Subsequently, the extracellular solution was replaced with solution one containing the NaCl by perfusion, and the I-V curve was recorded at least 1 min after the perfusion. The permeabilities to K^+^, Cs^+^, and Ca^2+^ were expressed as the ratios relative to the permeability of Na^+^ and were calculated according to Hille, 1972 (38). For P_K_ and P_Cs_, Ca^2+^ and Mg^2+^ were excluded from the permeability calculations due to their very low permeability and low concentration used (1 mM). For the analysis of permeability, reversal potentials were corrected by liquid junction potentials estimated with Clampex9.2.

For permeability of K^+^ and Cs^+^, the ratio of the permeability was calculated according to the following equation:

(5) $$\frac{P_{X}}{P_{Na}}=\frac{\alpha_{Nae}}{\alpha_{Xe}}\left[\exp\left(\frac{{\Delta E}_{rev}}{RT/F}\right)\right]$$

where ΔE_rev_, α_*x*_, R, T, and F are the reversal potential, effective activity coefficients for cation x (e, external), the universal gas constant, absolute temperature, and the Faraday constant, respectively. RT/F = 25.4 mV. The effective activity coefficients (α_*x*_) were calculated using the following equations:

(6) $$\alpha_{\chi }=\gamma_{\chi }\left[X\right]$$

where γ_*x*_ is the activity coefficient and [X] is the concentration of the ion. For calculations of membrane permeability, activity coefficients (γ) was calculated using the Debye-Hückel equation: 0.74, 0.72, and 0.69 correspond to Na^+^, K^+^, and Cs^+^, respectively.

### Data analysis

Data were analyzed PatchMaster v2x73 (HEKA Elektronik), IgorPro6 (Version 6.1.0.9, WaveMetrics, Lake Oswego, OR, USA), Prism 9.5 (Version 9.5.0, GraphPad Software) and Office Excel 2010 (Version 14.0.4760.1000, Microsoft). All values are shown as mean ± SD, and n represents the number of cells examined. An unpaired two-tailed test was used to assess the difference between two groups. One-way ANOVA and two-way ANOVA were used to assess the difference between multiple groups. In figure legends, the *p* values are also shown. Statistical analyses were performed with Prism 9.5 software.

## Data availability

All data are available contained in the main text.

## Conflict of interests

The authors declare that they have no conflicts of interest with the contents of this article.
